# ﻿ *Carexmalipoensis* (Cyperaceae), a new species from southeast Yunnan, China

**DOI:** 10.3897/phytokeys.188.75401

**Published:** 2022-01-07

**Authors:** Yuan-Yuan Li, Ya-Ping Chen, Li-Qiong Jiang, En-De Liu, Yuan Luo, Hua Peng

**Affiliations:** 1 CAS Key Laboratory for Plant Diversity and Biogeography of East Asia, Kunming Institute of Botany, Chinese Academy of Sciences, Kunming 650201, China Kunming Institute of Botany, Chinese Academy of Sciences Kunming China; 2 University of Chinese Academy of Sciences, Beijing 100049, China University of Chinese Academy of Sciences Beijing China; 3 School of Ecology and Environmental Sciences & School of Life Sciences, Yunnan University, Kunming 650091, China Yunnan University Kunming China

**Keywords:** *Carex* sect. *Euprepes*, *Carextrichophylla*, morphology, taxonomy

## Abstract

*Carexmalipoensis*, a new species from southeast Yunnan, China, is here described and illustrated. It is morphologically similar to *C.trichophylla* in sect. Euprepes, but differs from it by its longer inflorescences and peduncles, pendulous spikes, hispidulous female glumes, densely hispidulous utricles, and longer nutlets.

## ﻿Introduction

As one of the largest angiosperm genera, *Carex*Linnaeus (1753) (Cyperaceae) comprises an extremely high diversity of about 2000 species (Roalson et al. 2021; WCSP 2021). The genus has a Cosmopolitan distribution. It was placed within the tribe Cariceae and divided into four subgenera by Kükenthal (1909): subg. Psyllophora (Degland 1828) Petermann (1849), subg. Vigneastra (Tuckerman 1843) Kükenthal (1899), subg. Vignea (P. Beauv. ex Lestiboudois 1819) Petermann (1849) and subg. Carex. This classification of Kükenthal had been widely adopted by subsequent researchers for a long time. Recent molecular phylogenetic studies revealed that Cariceae is a natural group, and the previously recognized genera of this tribe, i.e., *Cymophyllus* Mack. ex Britton & A.Br., *Kobresia* Willdenow, *Schoenoxiphium* Nees von Esenbeck, and *Uncinia* Persoon, should be merged into *Carex* (Yen and Olmstead 2000; Global *Carex* Group 2015; Starr et al. 2015; Léveillé-Bourret et al. 2018; Martín‐Bravo et al. 2019; Villaverde et al. 2020; Larridon et al. 2021; Roalson et al. 2021). A framework of the combined giant genus was urgently needed to increase our understanding of *Carex*. In order to solve this problem, Villaverde et al. (2020) conducted a robust phylogeny of *Carex* based on molecular data (308 nuclear exon matrices, 543 nuclear intron matrices and 66 plastid exon matrices) and six clades were recognized. Accordingly, Villaverde et al. (2020) proposed an updated infrageneric classification of *Carex* and classified it into six subgenera: subg. Siderosticta M.J. Waterway, subg. Carex, subg. Euthyceras., subg. Psyllophorae, subg. Uncinia (Pers.) Peterm. and subg. Vignea. However, the classification within the subgenera still remained unresolved, so a more systematic and friendly infrageneric classification system was required. A framework infrageneric classification of *Carex* was proposed recently which divided *Carex* into 62 formally named Linnean sections and 49 informal groups based on the current phylogenetic knowledge of *Carex* (Roalson et al. 2021).

A total of 527 *Carex* were recorded in Flora of China (Dai et al. 2010), the Catalogue of Life China (Chen and Zhang 2018) recorded 593 species, which represents the most complete and update list of the genus in China. The number of species of *Carex* continues growing in China as more new species have been reported in recent years (Lu and Jin 2018; Zhang et al. 2018; Jin et al. 2020; Lu et al. 2020; Yang and Liu 2020).

During our field investigations between 2016 and 2018, we collected specimens of an unknown species of *Carex* in Malipo County, southeast Yunnan Province, China. After careful morphological studies, examination of herbarium specimens and relevant literature, we concluded that it can be assigned to Carexsect.Euprepes based on a combination of some morphological characters: cauline leaves well-developed; leaf blades and involucral bracts elliptic to linear-elliptic, with prominent transverse veins; complex branched inflorescence; spikes androgynous; and presence of utriculiform cladoprophylls (Jiménez-Mejíias et al. 2016) at the base of spikes. Molecular phylogenetic studies indicated that C.sect.Euprepes belongs to the core *Carex* clade (Starr et al. 2015; Villaverde et al. 2020), and the most recent infrageneric classification framework placed it with the Indica Clade together with species traditionally placed in sections *Euprepes* and *Mapaniifoliae* (Roalson et al. 2021). Consisting of seven species, sect. Euprepes are restricted to South and Southeast Asia (Nelmes 1955). Only one species of the section, *C.zizaniifolia*Raymond (1959), was previously reported from China, distributed in southeast Yunnan Province (Dai et al. 2010; Chen and Zhang 2018). However, after our research on this section, we conclude that our new collections are different from all known species and represent a species new to science. We describe and illustrate it here below.

## ﻿Materials and methods

The new species was compared morphologically with specimens of other taxa of Carexsect.Euprepes from the following public herbaria A, BM, E, GH, HNU, IBSC, K, KUN, HUH, MO, MT, NY, P, PE, and US [acronyms follow Theirs (continuously updated)] as well as our new collections across China (especially with material collected from southeast Yunnan and neighboring area; herbarium specimens kept in KUN). Meanwhile, protologues and other related taxonomic literature were collated and reviewed. The characters’ data come from specimens measurements and the prologue (*C.atrivaginata* Nelmes and *C.tricophylla*Nelmes (1955), *C.euprepes* Nelmes and *C.laosensis*Nelmes (1939), *C.tavoyensis*Nelmes (1948), *C.zizaniifolia* Raymond and *C.poilanei*Raymond (1959)). The terminology used by Kükenthal (1909) for the morphological description of *Carex* species was adopted here. The distribution of the new species was compiled from the herbarium specimen records and our own collections, and shown on the distribution map.

## ﻿Results

A detailed morphological comparison of the potential new species and the seven species of C.sect.Euprepres is summarized in Table 1. Morphologically, the new species is most similar to *C.tricophylla* but can be distinguished by the characteristics of culms (70–105 cm long, 2–4 mm thick, sides ribbed in the new species vs. culms 45–65 cm long, 1–1.5 mm thick, sides concave in *C.tricophylla*); leaf sheaths (1.5–7 cm long vs. 1.2–2 cm long); inflorescences (20–45 cm long, peduncles up to 9 cm long vs. 3–15 cm long, more or less exserted), spikes (15–33 mm long, pendulous vs. 7–10 mm long, erect), female glumes (hispidulous vs. glabrous), utricles (densely hispidulous vs. glabrous below, adpressed-hispid above), and nutlets (4–4.5 mm long vs. 2.25–2.5 mm long). The new taxon can be distinguished from all the other seven species in this section by their culms (length, cross-sectional shape, sides and indumentum); leaves (shape, transverse veins), sheath (length, indumentum and the appendage of mouth); inflorescence bigger (20–45 × 3–5 cm in the new taxa vs. 3–25 × 1–5 cm in the other species) or with different shape (oblong in the new taxon vs. oblong, narrowly oblong or triangular-ovate in other species); peduncles longer (up to 9 cm long in the new taxon vs. scarcely or slightly exserted in other species; only *C.atrivaginata* has relative long peduncles not exceeding 5 cm); and the female glumes, utricles and nutlets (differences in shape, size and indumentum).

**Table 1.** Morphological comparisons between *Carexmalipoensis* and species in C.sect.Euprepes.

Characters	*C.malipoensis*	*C.atrivaginata*	*C.euprepes*	*C.laosensis*	*C.poilanei*	*C.tavoyensis*	*C.tricophylla*	*C.zizaniifolia*
**Culm**								
Length	70–105 cm	65 cm	70 cm	unknown	70–105 cm	unknown	45–65 cm	60–70 cm
Thick	2–4 mm	3 mm	3 mm	2 mm	2–3 mm	2–2.5 mm	1–1.5 mm	2 mm
Transverse angle	obtuse	acute	obtuse	obtuse	acute	acute, obtuse to subacute	obtuse	obtuse
Sides	ribbed	concave	concave	concave	concave	ribbed, slightly twisted	concave	concave
Indumentum	hispidulous	glabrous	glabrous	glabrous	glabrous	glabrous	hispidulous upward	glabrous
**Leaf**								
Number	3–5	7	4–6	1–4	6–8	unknown	6 or more	8
Shape	elliptic	narrowly linear-lanceolate	elliptic	narrowly linear-lanceolate	narrowly linear-lanceolate	Linear-elliptic	elliptic, apex attenuated	narrowly elliptic or lanceolate
Length	12–28 cm	13–16 cm	17–24 cm	20–30 cm	18–30 cm	25–28 cm	12–18 cm	15–25 cm
Width	2–3 cm	1–1.3 cm	3–4.5 cm	8–15 mm	1–2 cm	2.3–3 cm	1.2–2 cm	2–2.5 cm
Indumentum	glabrous, sparsely hispidulous on undersurface midrib	glabrous, apex hispidulous	scabrid along veins undersurface	glabrous	glabrous	scabrid along veins undersurface	scabrid along veins undersurface	scabrid along veins
**Leaf sheath**								
Length	1.5–7 cm	1.5–5 cm	1–2 cm	1.5–2 cm	2–5 cm	unknown	1.5–2 cm	1.4–2 cm
Indumentum	hispidulous	glabrous	glabrous below, hispidulous above	hispidulous	glabrous	glabrous	hispidulous	glabrous
Appendage of sheath mouth	not developed	not developed	prominent	small	small	small	prominent	small
**Inflorescence**								
Shape	oblong	oblong	narrowly oblong	narrowly oblong	triangular-ovate	narrowly oblong	narrowly oblong	narrowly oblong
Length	20–45 cm	8–10 cm	10–22 cm	3.5–13.5 cm	12–20 cm	15–25 cm	3–15 cm long	5–6 cm
Width	3–5 cm	2–5 cm	3–4 cm	1–2.5 cm	3–6 cm	1–2 cm	1–2 cm	1–1.5 cm
Peduncles	exserted	exserted	scarcely or slightly exserted	scarcely exserted	exserted	scarcely exserted	exserted	scarcely exserted
**Spikes**								
Length	15–30 (45) mm	10–25 mm	8–10 mm	5 mm	8–18 mm	5–9 mm	7–10 mm	5–6 mm
Male part vs female part	much longer	much longer	equal	longer	much longer	slightly longer	longer	slightly longer
**Utricles**								
Shape	ovate-elliptic	ovate-elliptic	elliptic	broadly ellipsoid or ellipsoid-obovoid	ovate or rhomboid-ovate	ellipsoid or obovoid-ellipsoid	obovoid or ellipsoid-obovoid	ovate-elliptic
Length	6–7.5 mm	6–7.5 mm	4–4.5 mm	4–4.5 mm	4–5 mm	4–5 mm	6–6.5 mm	ca. 3 mm
Indumentum	densely hispidulous	densely hispidulous	glabrous, margins hispidulous	glabrous	unknown	glabrous below, adpressed-hispid above	glabrous below, adpressed-hispid above	densely hispidulous
**nutlets**	ca. 4 mm	ca. 4 mm	2.25–2.5 mm	2.25–2.8 mm	unknown	ca. 2.3 mm	2.25–2.5 mm	immature

The characters marked “unknown” for the missing description of the protologue or the original materials incomplete.

## ﻿Taxonomy

### ﻿ Carex malipoensis

Taxon classificationPlantaePoalesCyperaceae

Yuan Y. Li & H. Peng sp. nov.

AD0440CA-F598-5CBC-BEA8-277C16DBDDEF

urn:lsid:ipni.org:names:77235050-1

Figures 1 , 2

#### Type.

China. Yunnan Province, Malipo County, Mengdong Village, Xiangchunping, 22°54'36.77"N, 104°38'54.09"E, alt. 1850 m, 5 December 2016, E.D. *Liu et al. LiuED6425* (holotype: KUN! Barcode 1433368; isotype: KUN! Barcode 1347669).

**Figure 1.**
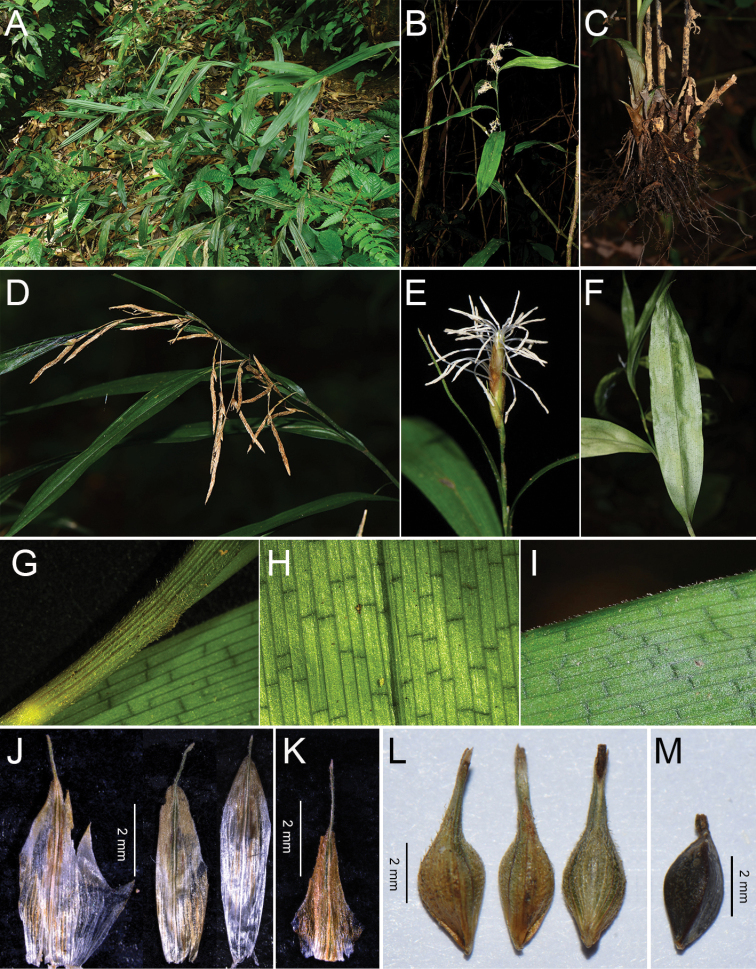
Morphology of *Carexmalipoensis***A** habitat **B** plant **C** rhizome **D** inflorescence **E** spike **F** leaf blade **G** sheath **H** the transverse veins **I** leaf margin **J** male glumes **K** female glume **L** utricles **M** achene. Scale bars: 2 mm. Photographed by X.X. Zhu (**A, D, E***LiuED6425*, *LiuED5912* KUN), Y.P. Chen (**B, C, F, I***Y.P. Chen & L.Q. Jiang MLP01* KUN), and Y. Y. Li (**G, H, J–K***Y.P. Chen & L.Q. Jiang MLP01* KUN; **L, M***LiuED6425* KUN).

#### Diagnosis.

The new species is most similar to *C.trichophylla*Nelmes (1955), but differs in inflorescences 20–45 cm long (vs. shorter than 15 cm in *C.trichophylla*), peduncles up to 9 cm long (vs. more or less exserted in *C.trichophylla*), spikes pendulous (vs. erect in *C.trichophylla*), female glumes hispidulous (vs. glabrous in *C.trichophylla*), utricles densely hispidulous (vs. glabrous below, adpressed-hispid above in *C.trichophylla*), and nutlets 4–4.5 mm long (vs. 2.25–2.5 mm long in *C.trichophylla*).

**Figure 2.**
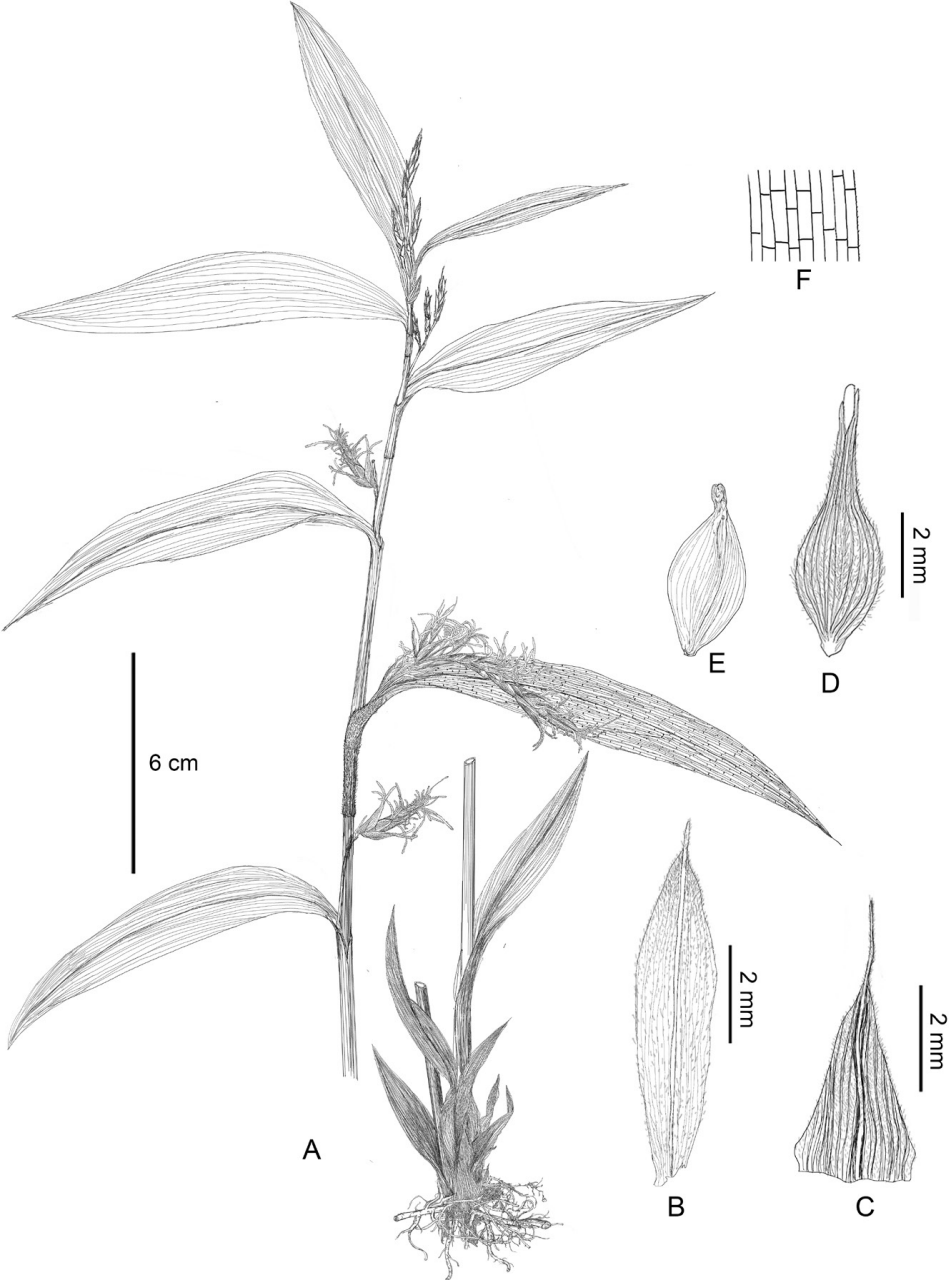
Line drawing of *Carexmalipoensis***A** habit **B** male glume **C** female glume **D** utricles **E** achene **F** part of blade, show the transverse veins. Scale bars: 6 cm (**A**); 2 mm (**B–E**). Drawn by Yuan Luo from the type specimen.

#### Description.

Rhizomes elongate. Culms tufted, 70–105 cm long, 2–4 mm in diam, obtusely trigonous, hispidulous, basal sheaths dark brown. Leaves 3–5, basal and cauline, loosely arranged; leaf blade elliptic, 12–28 × 2–3 cm, transverse veins prominent, margin hispidulous-villous, base round to cuneate, apex acute, greyish green when dried; sheaths 1.5–7 cm long, hispidulous, mouth hairy, not developed into prominent appendage. Involucral bracts leaf-like, longer than inflorescence, sheathing; panicles 20–45 × 3–5 cm, with 12–16 branches, single or binate; peduncles up to 9 cm long, reduced toward apex, tenuous, glabrous or slightly pubescent; inflorescence axes sharply trigonous, hairy on edges; bractlets glumiform, ca. 5 mm long, apex awned, awns 4–5 mm long; cladoprophylls utriculiform, 3–4 mm long; spikes bisexual, androgynous, 15–30 (–45) mm long; male part of spike much longer than female part; male part densely many flowered, ca. 2.5 mm wide; female part fewer flowered, 3–4.5 mm wide; male glumes oblong-lanceolate, 6–9 × 1.5–2 (–3.2) mm, awned; female glumes oblong-lanceolate, 3.8–4.2 mm long, pale brown, green at middle, apex acute, midrib extending into a scabrid awn. Utricles ovate-elliptic, 6–7.5 mm long, green to brown, veined, densely hispidulous, apex attenuating into a long beak, ca. 3 mm long, orifice oblique. Nutlets ca. 4 × 1.8–2 mm, dark brown, obovate-elliptic, trigonous. Stigmas 3.

#### Phenology.

Flowering from November to December, and fruiting in May.

#### Distribution.

**Figure 3.**
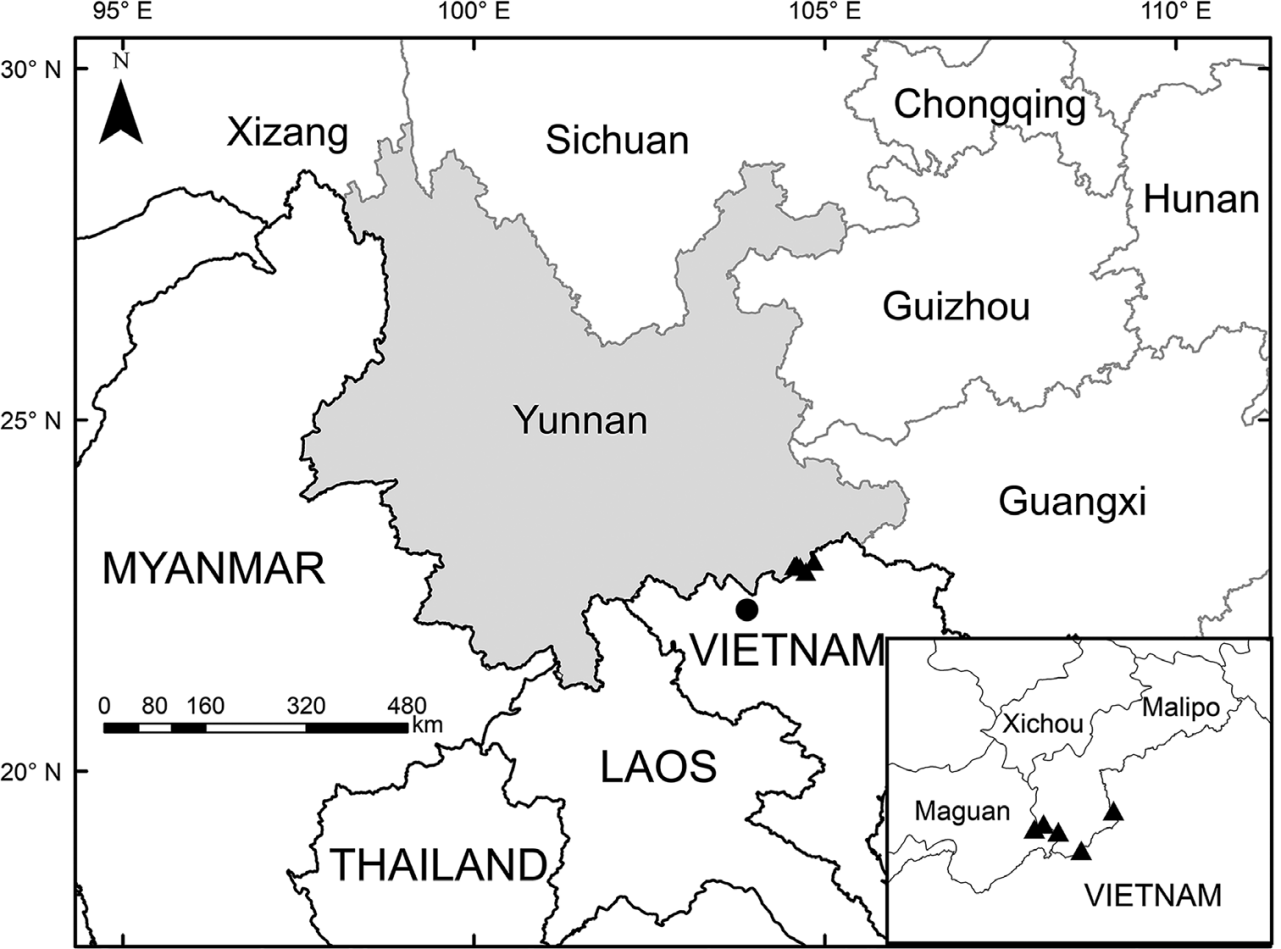
Distribution map of *Carexmalipoensis* (▲) and *C.trichophylla* (•).

The new species is currently known from Malipo County in southeast Yunnan at the Sino-Vietnamese border (Fig. 3).

#### Habitat.

The new species usually grows in evergreen broad-leaved forests at altitudes of 1100–1850 m.

#### Etymology.

The specific epithet refers to Malipo County of Yunnan Province, China, from where the type specimens were collected.

#### Common name (assigned here).

Ma Li Po Tai Cao (麻栗坡薹草; Chinese name).

#### Additional specimens examined (paratypes).

China. Yunnan: Maguan County, Ching-kou Loa-chün-shan, 7 December 1947, *K.M. Feng 13677* (KUN0368409; KUN1263725); Malipo County, Mengdong Village, 4 December 2016, *E.D. Liu et al. LiuED 6336* (KUN1433717), *LiuED6403* (KUN1433131); Malipo County, Laojunshan, Bailingyan, 13 May 2017, *E.D. Liu et al. LiuED5912* (KUN1340680); Malipo County, Tianbao Town, Bajiaoping Village,1 December 2018, *Y.P. Chen & L.Q. Jiang MLP01* (KUN).

#### Specimens examined of other species.

***Carexatrivaginata***: Vietnam. Chapa: *Pételot*, *3179* (P00277787, P00277788); *E. Poilane*, *27084* (MT00072452, MT00072458); ***C.euprepes***: LAOS. Tawieng, Chiengkwang: 2 April, 1932, *Kerr*, *20927* (BM001172101, K000291207, K000291208, K000291210, NY04059693, P00282617); ***C.laosensis***: Laos. Pak Munung, Wieng chan: 22 April, 1932, *Kerr*, *21202* (K000291209, K000291210, P00284722); ***C.poilanei***: LAOS. Phong Saly: 6 September, 1941, *E. Poilane*, *25984* (MT00117677); *E. Poilane*, *32994* (MT00072475); ***C.tavoyensis***: Myanmar. Padachaung, Tavoy. 3 April, 1921, *P.T. Russell*, *1935* (K000999214); ***C.trichophylla***: Vietnam. Chapa (Fig. 3): 1 July, 1930, *Pételot*, *5325* (GH00027549, P00302178, P00302179, P00302180, US00087306); ***C.zizaniifolia***: China. Yunnan, Pingbian: 1934, *H. T. Tsai 62809* (A00027543, IBSC0653006, KUN0368701, PE00030290).

#### Conservation status.

The new species is currently known from Maguan and Malipo Counties in Yunnan, China. Only six collections have been recorded since 1947. It may be classified as Endangered (EN) or Vulnerable (VU) according to the IUCN Red List criteria(IUCN 2012). However, collections of *Carex* are often deficient, and a solid suggestion is needed based on a comprehensive investigation about the new species. Therefore, we suggest to characterize the conservation status of *C.malipoensis* as Data Deficient (DD) at present.

## ﻿Discussion

In comparison with other species of C.sect.Euprepes, *C.malipoensis* is morphologically most similar to *C.trichophylla*. Both species have obtusely trigonous culms and elliptic and subpetioleate leaf blades, inflorescences big and loose, and utricles longer than 6 mm, all characters that differ from all the remaining species of C.sect.Euprepes. Despite their shared similarities, *C.malipoensis* can be distinguished from *C.trichophylla* in the length of inflorescences and peduncles, and indumentum of sheath and utricles. Specifically, *C.malipoensis* has much longer culms and spikes, and larger leaves, panicles, and achenes compared with that of *C.trichophylla*. Moreover, the female glumes and utricles are hispidulous in *C.malipoensis*, but glabrous in *C.trichophylla*.

## Supplementary Material

XML Treatment for Carex malipoensis
